# Rotational Dynamics of the Distal Tibiofibular Joint After Operative Treatment of Ankle Fractures With Syndesmosis Injury

**DOI:** 10.1177/10711007251392222

**Published:** 2025-12-08

**Authors:** Ristomatti Lehtola, Tero Kortekangas, Harri Pakarinen, Iikka Lantto, Pasi Ohtonen, Jaakko Niinimäki, Hannu-Ville Leskelä

**Affiliations:** 1 Department of Surgery, Division of Orthopaedic and Trauma Surgery, Oulu University Hospital, Finland; 2 Medical Research Center Oulu, University of Oulu, Finland; 3 Pihlajalinna Hospital Oulu, Finland; 4 Research Service Unit, Oulu University Hospital, Finland; 5 Translational Medicine Research Unit, University of Oulu, Finland; 6 Department of Radiology, Oulu University Hospital, Finland

**Keywords:** ankle, fracture, syndesmosis

## Abstract

**Background:**

Syndesmosis injury healing remains poorly understood, despite its high prevalence in ankle fractures. Unstable syndesmosis is commonly addressed with either syndesmosis screw (SS) or suture button (SB) fixation, and up to 20% of operated ankle fractures may require some form of syndesmosis fixation. However, in biomechanical studies no repair technique fully restores the preinjury rotational stability or the anatomical alignment of the tibiofibular joint.

**Methods:**

In a study of 39 patients with operatively treated supination external-rotation type 4 and pronation external-rotation type 4 ankle fractures and fixation of an unstable syndesmosis, weightbearing cone beam computed tomography with rotational stress was performed on both ankles at a mean follow-up of 7.8 (range, 6.2-10.3) years to evaluate tibiofibular syndesmosis dimensions and fibular rotation. Sagittal translation of the fibula (ST), anterior width (AW) and posterior width (PW) of the syndesmosis, tibiofibular clear space (TFCS), and fibular rotation (RO) were measured in neutral position and in maximal internal and external rotation. Mean change in measurements between maximal rotations were calculated to represent range of motion of the fibula under rotational stress.

**Results:**

Twenty-six patients had screw fixation (SS) and 13 had suture button (SB) fixation of the syndesmosis. Eight SSs had been removed and 3 were broken. No SBs had been removed. The mean Olerud-Molander Ankle Score was 84.7 (SD 20.3). Fibular rotation demonstrated a mean difference of 2.7 degrees (95% CI, 1.3-4.1; *P* < .05) compared with the patient’s non-injured ankle. Other measurements showed no significant differences; however, we lacked statistical power to detect significant changes in ST, AW, PW, and TFCS.

**Conclusion:**

Excess fibular rotation persists after healing of ankle fractures with fixed unstable syndesmosis. However, clinical relevance remains unclear and should be explored with larger patient groups.

**Level of Evidence:** Level III, cohort study.

## Introduction

The natural course of syndesmosis injury healing is unknown, although syndesmosis injuries are common in ankle fractures.^10,30^ When syndesmosis instability persists following bony fixation, it is commonly addressed with either syndesmosis screw (SS) or suture button (SB) fixation.^31,36,38^ Up to 20% of operated ankle fractures may require some form of syndesmotic fixation.^37^ Functional outcomes are comparable between patients treated with syndesmotic screws (SS) and suture-button (SB) fixation.^11,15,34^ Biomechanical studies indicate that no repair technique fully restores rotational stability or the anatomical alignment of the tibiofibular joint to its preinjury state.^6,21,32^ To date, no clinical studies have been published on rotational dynamics of the distal tibiofibular joint following the healing of syndesmosis injuries with concomitant ankle fracture.

Computed tomography (CT) of both ankles is the preferred imaging modality for evaluating the syndesmosis integrity.^5,17,19,20^ However, conventional CT performed in a supine position does not provide information about syndesmosis stability or functional behavior under physiological loading conditions.^1,3,17,19,20,35^ Given that external rotation of the upper ankle joint is the predominant mechanism of injury to the distal syndesmosis,^14,36^ the most biomechanically accurate method for detecting residual instability of the distal tibiofibular joint would incorporate rotational stress during weightbearing radiographic imaging.^2-4,13,29,33^

Our previous study has provided reference values for normal syndesmosis using weightbearing cone beam computed tomography (WBCT) and rotational stress of the ankle joint.^19^

The aim of this study was to evaluate the rotational dynamics of the distal tibiofibular joint after operative treatment and healing of a syndesmosis injury using WBCT under maximal rotation of the injured ankle.

## Methods

The study population consisted of patients from 2 of our previous trials involving operatively treated ankle fractures and syndesmosis fixation.^11,12,15,16,27^ All patients had a supination external-rotation type 4 (SE4) or pronation external-rotation type 4 (PE4) ankle fracture and pre- or intraoperative evidence of syndesmosis instability, based on a plain radiograph or a manual external rotation stress test (ER test).^10-12,15,16,27^ In the first trial, after fixation of bony injuries, manual 7.5-Nm ER test was done to assess syndesmosis stability. More than 2-mm side-to-side difference in tibiotalar or tibiofibular clear spaces was considered unstable. SE4 patients with unstable syndesmosis were randomized to receive either 3.5-mm fully threaded tricortical SS or no syndesmosis fixation.^12,16,27^ In the second trial, PE4 patients with unstable syndesmosis were treated with either SS (3.5-mm fully threaded tricortical) or SB (Tightrope; Arthrex, Naples, FL), depending on randomization.^11,15^ All patients had clearly unstable ankle mortise on plain radiograph preoperatively or unstable ER test after malleolar fixation as described above.

At the planned final follow-up visit of the forementioned trials, we conducted WBCT (Planmed Verity Extremity; Planmed Oy, Helsinki, Finland) under rotational stress of both ankles.

Patients with bilateral ankle fractures, pathologic fractures, concomitant tibial shaft fractures, previous significant injury or a fracture of either ankle, significant peripheral neuropathy, soft tissue infection in the region on either injured ankle, or inability to complete the study protocol were excluded from the forementioned trials.

**Figure 1.**
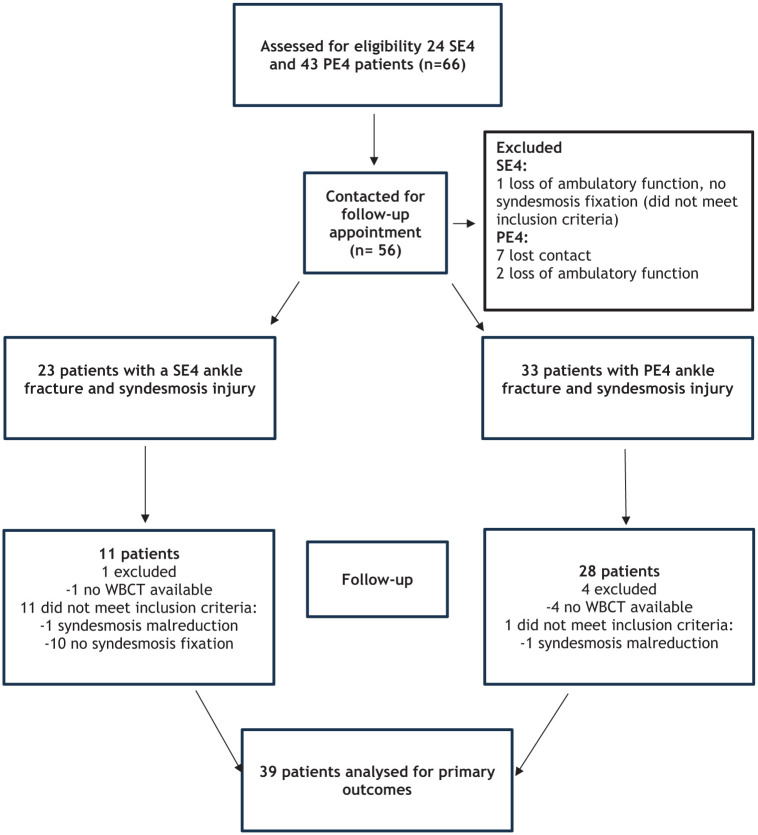
Study flowchart.

For the present study, only patients with syndesmosis fixation and a reduced syndesmosis were included, omitting those without fixation or with malreduction to minimize potential confounding factors. Furthermore, as WBCT was performed at the final follow-up of the previous trials, some patients declined to undergo the scan and were therefore not part of the analysis. Consequently, the present cohort comprised only patients treated with SS or SB fixation and a reduced syndesmosis (Figure 1).

Follow-up visits were carried out at Oulu University Hospital between December 2017 and January 2019. The local ethics review board approved the study plan, and all patients provided written informed consent. At the follow-up visit, patients completed the Olerud-Molander Ankle Score (OMAS) to assess ankle function.^26^ The OMAS is a validated, condition-specific, patient-reported outcome measure for ankle fracture symptoms (range, 0-100), with higher scores indicating better function and fewer symptoms.^25,26^ The study was conducted in accordance with the Declaration of Helsinki.

The prespecified primary outcomes of the study were the difference between the mean changes in sagittal translation of the fibula (ST), anterior width (AW) and posterior width (PW) of the syndesmosis, tibiofibular clear space (TFCS), and fibular rotation (RO) between injured and healthy ankles during upright weightbearing ankle rotation from maximal internal rotation to maximal external rotation.

### Radiographic Technique

Patients stood barefoot on a designated platform, maintaining a natural stance without rotation of the lower leg or pelvis and with neutral pressure on the foot. The other foot was lightly rested on the gantry of the WBCT device. For external rotation imaging, patients were instructed to maximally rotate their entire body inward, which positioned the tibia in internal rotation and the talus in external rotation. Throughout, the medial side of the sole and the distal metatarsal line of the foot remained in contact with the platform. For internal rotation imaging, patients maximally rotated their entire body outward, positioning the tibia in external rotation and the talus in internal rotation. During this maneuver, the lateral side of the sole and the distal metatarsal line maintained contact with the platform. Imaging was performed when patients experienced discomfort in the ankle, knee, or hip, or when the distal metatarsal sole of the foot began to lift from the platform, to ensure that maximal degree of talar rotation was achieved. We did not measure the amount of torque. However, in a previous study using the same imaging protocol, a mean rotational force applied 15 cm distal to the center of rotation of the ankle was 200 N resulting in a moment of 30 Nm.^18,19^

All the studies were supervised by senior orthopaedic trauma surgeons or by an intern who had completed university hospital trauma training. The imaging protocol was identical to that reported previously by Lepojärvi et al.^17,19^

### Measurements

CT data were analyzed on a clinical workstation, using bone window reformations. Measurements were obtained 10 mm above the tibial subchondral bone from reformatted axial CT planes that were aligned exactly parallel to the distal tibial plafond.^7,8,10,17,19^

**Figure 2.**
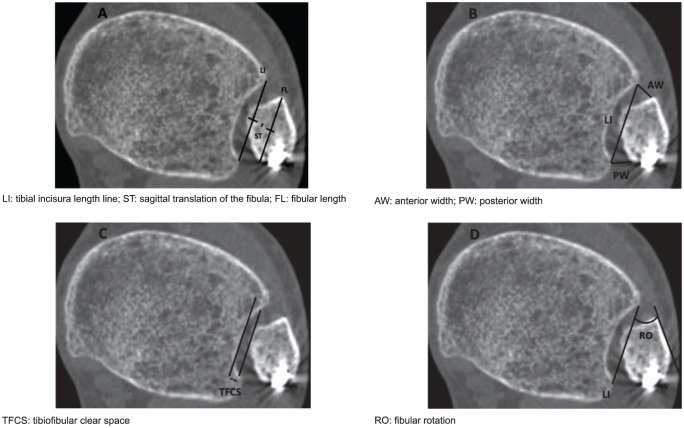
(A-D) Radiographic measurements.

First, the tibial incisura length line (LI) was drawn tangentially along the most prominent parts of the anterior and posterior tibial tubercles (Figure 2A). ST was defined as the difference between the midpoints of the fibular length (FL) and the LI (Figure 2A). To determine AW and PW of the syndesmosis, lines were drawn from the LI to the most proximal part of the fibula at the intersections with the tibial tubercles (Figure 2B). TFCS was measured as the true distance between the fibula and the deepest point of the tibial incisura, tangential to the edges of the incisura (Figure 2C). RO was calculated as the angle between the LI and the anterolateral cortex of the fibula (Figure 2D). Measurements were made to 0.1-mm and 1-degree accuracy.

These same measurements were taken in neutral, internal, and external rotation positions. Mean change of ST, AW and PW, TFCS, and RO between positions were calculated, indicating the range of motion of the fibula in upright weightbearing ankle rotation.

All measurements were performed by a single investigator (R.L.). The measurement protocol was identical to that reported previously by Lepojärvi et al.^17,19^ Previously reported ICC values for interobserver reliability were 0.73-0.92 and for intraobserver reliability 0.75-0.99, showing excellent reliability of the method.^19^

The mean width of the syndesmosis in neutral position was calculated as described previously by Mukhopadhyay et al^24^ in 2011 as $\frac{(\;{\mathrm{AW}}_{\mathrm{injured}\;\mathrm{ankle}}\;-\;\;{\mathrm{AW}}_{\mathrm{uninjured}\;\mathrm{ankle}})\;+\;{(}_{\mathrm{PW}\;\mathrm{injured}\;\mathrm{ankle}}\;\;-\;\;\;{\mathrm{PW}}_{\mathrm{uninjured}\;\mathrm{ankle}})}{2}.$

A side-to-side difference greater than 2 mm in the mean width of the syndesmosis was considered malreduced.^24^

### Statistical Analysis

All analyses were performed using IBM SPSS Statistics for Windows (version 29.0; IBM Corp, Armonk, NY). Summary measurements are presented as mean with SD unless otherwise stated. Student *t* test for paired samples was used to compare continuous variables. For all continuous outcomes, differences between injured and non-injured ankles are reported with 95% CIs. Categorical variables are analyzed using Pearson χ^2^ test or Fisher exact test. Two-tailed *P* values are reported. *P* value <.05 was considered statistically significant. Given the large number of dependent comparisons, the Bonferroni method was used to correct for multiple testing.

## Results

One SE4 patient and 1 PE4 patient, both with SS, were excluded from the analysis because of syndesmosis malreduction. The analysis included 11 patients with SE4 ankle fractures and 28 patients with PE4 ankle fractures. Of these, 26 patients had SS and, 13 patients had SB. Eight SSs had been removed and 3 were broken. No SBs had been removed. Mean OMAS was 84.7 (SD 20.3) (Table 1). The mean follow-up time was 7.8 (range, 6.2-10.3) years (Table 1). There were no significant differences between patients included in the study and those lost to follow-up or with insufficient imaging data (Supplementary Table S1).

**Table 1.** Baseline Characteristics.

Characteristic	Value
Fracture type, n	
Supination external-rotation type 4	11
Pronation external-rotation type 4	28
Follow-up time, y, mean (range)	7.8 (6.2-10.3)
Age at follow-up, y, mean (range)	52.8 (27.6-80.5)
Fracture anatomy, n	
Fibular fracture	39
Medial malleolus fracture	12
Deltoid ligament rupture	27
Posterior malleolus fracture	12
Trimalleolar fracture	8
Medial malleolus fixation	9
Posterior malleolus fixation	1
Syndesmosis fixation, n	39
Syndesmosis screw fixation	26
Suture button fixation	13
Implant removal, n	8
Syndesmosis screw fixation	8
Suture button fixation	0
Broken syndesmosis screw	3
Mean Olerud-Molander Ankle Score (SD)	84.7 (20.3)

Fibular rotation showed a mean difference of 2.7 degrees (95% CI, 1.3-4.1; *P* < .05) (Table 2). According to our post hoc power analysis, we had 93% power to detect a significant difference in fibular rotation at the 5% significance level (group size 39, alpha = 0.05, power = 0.80). The post hoc power analysis indicated a lack of statistical power to detect significant differences in ST, AW, PW, and TFCS, as suggested by the absence of statistically significant differences (Supplementary Table S2).

**Table 2.** Mean Change in Syndesmosis Measurements Between Maximal Rotations of the Ankle and Mean Differences Between the Injured and Non-injured Ankles of the Same Patient.

	Injured Ankle, Mean (SD)	Non-injured Ankle, Mean (SD)	Mean Difference	95 % CI	P*
Sagittal translation, mm	1.3 (1.1)	1.1 (1.0)	0.2	−0.2 to 0.6	>.9
Anterior width, mm	−0.7 (0.9)	−0.7 (0.8)	0.1	−0.3 to 0.5	>.9
Posterior width, mm	1.0 (1.3)	1.1 (1.0)	−0.1	−0.6 to 0.5	>.9
Tibiofibular clear space, mm	−0.1 (0.5)	0.02 (0.7)	−0.1	−0.4 to 0.1	>.9
Fibular rotation, degrees	2.4 (4.1)	−0.2 (2.5)	2.7	1.3 to 4.1	<.05

* Bonferroni method used to correct for multiple comparisons.

Data for different fracture types are shown in Table 3. Statistical hypothesis testing between these subgroups were not performed because of the small subgroup sizes.

**Table 3.** Mean Change in Syndesmosis Measurements Between Maximal Rotations of the Ankle and Mean Differences Between the Injured and Non-injured Ankles of the Same Patient in Different Patient Groups.

Fracture Type	Syndesmosis Fixation	Measurement	Injured Ankle (SD)	Non-Injured Ankle (SD)	Mean Difference	95% CI of the Difference
SE4	Syndesmosis screw (n = 11)	Sagittal translation	1.2 (1.5)	1.3 (1.2)	−0.05	−1.0 to 0.9
		Anterior width	−0.2 (1.1)	−1.0 (0.9)	0.8	−0.1 to 1.6
		Posterior width	1.2 (1.4)	1.2 (1.4)	0.05	−0.8 to 0.9
		Tibiofibular clear space	0.1 (0.4)	−0.3 (0.6)	0.4	−0.1 to 0.9
		Fibular rotation	2.9 (4.6)	−0.7 (2.7)	3.6	0.1 to 7.1
PE4	Syndesmosis screw (n = 15)	Sagittal translation	1.2 (1.0)	0.9 (0.8)	0.3	−0.3 to 0.9
		Anterior width	−0.9 (1.0)	−0.6 (1.0)	−0.3	−1.0 to 0.4
		Posterior width	0.9 (1.1)	0.7 (0.6)	0.2	−0.5 to 0.9
		Tibiofibular clear space	−0.2 (0.4)	0.2 (0.4)	−0.4	−0.7 to −0.1
		Fibular rotation	1.9 (4.5)	−0.1 (2.8)	2.1	−0.4 to 4.5
	Suture button (n = 13)	Sagittal translation	1.4 (1.2)	1.1 (1.0)	0.3	−0.6 to 1.2
		Anterior width	−0.7 (0.5)	−0.6 (0.6)	−0.05	−0.4 to 0.3
		Posterior width	0.9 (1.5)	1.4 (0.9)	−0.4	−1.7 to 0.8
		Tibiofibular clear space	−0.2 (0.7)	0.1 (0.8)	−0.3	−0.8 to 0.2
		Fibular rotation	2.6 (3.3)	0.1 (2.1)	2.5	0.3 to 4.8

Abbreviations: SE4, supination external rotation type 4; PE4, pronation external rotation type 4.

## Discussion

In this paired, contralateral-controlled analysis of patients drawn from two prospectively followed trials, the primary finding was a mean 2.7-degree greater fibular rotation at maximal external rotation in the previously injured ankle compared with the contralateral side. No significant differences were observed between the injured and healthy ankles in other syndesmosis parameters (ST, AW, PW, TFCS). However, because non-rotational metrics (ST, AW, PW, TFCS) were underpowered, their null results should be interpreted cautiously.

Using the same method in healthy ankles, the mean rotation of the fibula in maximal ankle rotations is 3.2 degrees (SD 2.8, 95% CI 2.1-4.2).^19^ However, a significant difference is observed only in PW, with no fibular rotation difference between the ankles of the same individual.^19^ The clinical significance of the 2.7-degree difference in fibular rotation observed in our study remains unclear. As this difference is nearly as large as the normal variation in healthy ankles, the aim of the present study was not to assess functional outcomes. Investigating the effect of distal tibiofibular rotational dynamics on functional outcome should be the focus of future studies.

Moreover, biomechanical studies have consistently shown that no repair technique completely restores rotational stability and tibiofibular anatomic relationships of the preinjury state.^6,21,32^ Our findings extend these observations, demonstrating that this condition persists even after the healing of syndesmosis injuries.

Peiffer et al^29^ used WBCT and external rotation stress in a cohort of 21 patients with acute syndesmotic ligament injury confirmed by magnetic resonance imaging. Using automated 3-dimensional measurement models, they found an increase in the anterior tibiofibular distance and fibular rotation.^29^ Their measurements support our perspective that syndesmotic lesions should be assessed in a standing position under rotational stress using CT imaging of both ankles.^29^ Furthermore, the concept of applying external rotation stress in WBCT has been validated through studies involving cadaver models and healthy individuals.^3,17,19,20,33^

The use of a WBCT with rotational stress is a major strength of our study. Furthermore, the imaging and measurement protocol has been previously validated in healthy individuals, demonstrating excellent intra- and interrater reliability.^17,19,20^ Our mean follow-up period of 7.8 years provides sufficient time to account for the natural healing process of the syndesmosis. Most studies in the ankle fracture literature evaluate final functional outcomes at 2 years after trauma.^23^

Some limitations of this study warrant discussion. First, the cohort included patients treated with 2 different syndesmosis fixation methods, as well as 2 different fracture types. This resulted in small patient subgroups, necessitating the analysis of all patients as a single cohort, as subgroup analysis with these numbers would not be statistically meaningful. Additionally, the small subgroup size limits the interpretation of subgroup effects and may reduce condition-specific insights.

Torque was not measured in this cohort. We acknowledge that patient-driven maximal rotation introduces variability in the applied torque, which may influence the reproducibility and interpretation of the findings. Although discomfort is a subjective measure, we consider it a justified indicator of maximal individual rotation, as it allows each patient to safely reach their own physiological limit. It is also worth noting that individuals exhibit variation in upper ankle joint anatomy and function (eg, tibial torsion), and that foot morphology also differs between individuals. However, bilateral variation is usually minor. Consequently, the degree and strength of maximal rotation depend largely on individual anatomical characteristics. Thus, previous trials using the same imaging protocol demonstrated minimal bilateral variation between patients’ ankles and a torque of 30 Nm, supporting the notion that sufficient rotational force was achieved.^17-19^ Moreover, standardized external rotation stress tests have shown that a much lower torque—only 7.5 Nm—is sufficient to assess the stability of the ankle mortise.^9,22,28^ Although there were no statistically significant differences between the patients analyzed and those lost to follow-up, heterogeneity exists between these groups in terms of fracture types and fixation methods. This discrepancy may introduce bias; however, we believe its impact to be minimal. In the lost to follow-up group, 1 patient (7%) had an SE4 fracture, and 13 patients (93%) had a PE4 fracture, compared with the analyzed group, in which 11 patients (28%) had SE4 fractures and 28 (72%) had PE4 fractures (Supplementary Table S1). Since PE4 fractures involve more extensive syndesmosis injury,^11^ this difference between the groups might have resulted in an even greater disparity in fibular rotation had all patients been included. Mean OMAS of our patients was 84.7, indicating good functional outcome. However, we report OMAS and hardware removal data solely as clinical background information for our patient cohort. No comparisons were made because of the absence of a control group, and the main focus of this study is the rotational dynamics of the distal tibiofibular joint.

## Conclusion

This study demonstrates that excess rotational movement persists in the distal fibula after operative treatment of ankle fractures with syndesmosis fixation. However, clinical relevance remains unclear and should be explored with larger patient groups.

## Supplemental Material

sj-docx-2-fai-10.1177_10711007251392222 – Supplemental material for Rotational Dynamics of the Distal Tibiofibular Joint After Operative Treatment of Ankle Fractures With Syndesmosis Injury Supplemental material, sj-docx-2-fai-10.1177_10711007251392222 for Rotational Dynamics of the Distal Tibiofibular Joint After Operative Treatment of Ankle Fractures With Syndesmosis Injury by Ristomatti Lehtola, Tero Kortekangas, Harri Pakarinen, Iikka Lantto, Pasi Ohtonen, Jaakko Niinimäki and Hannu-Ville Leskelä in Foot & Ankle International

sj-docx-3-fai-10.1177_10711007251392222 – Supplemental material for Rotational Dynamics of the Distal Tibiofibular Joint After Operative Treatment of Ankle Fractures With Syndesmosis Injury Supplemental material, sj-docx-3-fai-10.1177_10711007251392222 for Rotational Dynamics of the Distal Tibiofibular Joint After Operative Treatment of Ankle Fractures With Syndesmosis Injury by Ristomatti Lehtola, Tero Kortekangas, Harri Pakarinen, Iikka Lantto, Pasi Ohtonen, Jaakko Niinimäki and Hannu-Ville Leskelä in Foot & Ankle International

sj-pdf-1-fai-10.1177_10711007251392222 – Supplemental material for Rotational Dynamics of the Distal Tibiofibular Joint After Operative Treatment of Ankle Fractures With Syndesmosis Injury Supplemental material, sj-pdf-1-fai-10.1177_10711007251392222 for Rotational Dynamics of the Distal Tibiofibular Joint After Operative Treatment of Ankle Fractures With Syndesmosis Injury by Ristomatti Lehtola, Tero Kortekangas, Harri Pakarinen, Iikka Lantto, Pasi Ohtonen, Jaakko Niinimäki and Hannu-Ville Leskelä in Foot & Ankle International
