# Supplementary material for: Rotational Dynamics of the Distal Tibiofibular Joint After Operative Treatment of Ankle Fractures With Syndesmosis Injury
Source: Foot Ankle Int. 2025 Dec 8;47(2):216–23. doi: 10.1177/10711007251392222 (PMC12882989; doi:10.1177/10711007251392222)
Supplement: sj-docx-2-fai-10.1177_10711007251392222 – Supplemental material for Rotational Dynamics of the Distal Tibiofibular Joint After Operative Treatment of Ankle Fractures With Syndesmosis Injury [file sj-docx-2-fai-10.1177_10711007251392222.docx]

**Supplementary table S1.** Comparative table summarizing baseline characteristics of patients included versus patients with syndesmosis fixation and lost to follow-up, excluded from the original trials^11,12, 15, 16, 25^ or without WBCT.

|  | **Patients analyzed**  **n=39** | **Lost to follow-up***  **n=14** | ***P*** |
| --- | --- | --- | --- |
| **Fracture type, n (%)** |  |  |  |
| Supination external-rotation type 4 | 11 (28) | 1 (7) | .1 |
| Pronation external-rotation type 4 | 28 (72) | 13 (93) |  |
| **Age at index trauma** | 45.0 (20-73.1) | 44.6 (19.7-78.7) | .9 |
| **Age at follow-up, mean, years (range)** | 52.8 (27.6–80.5) |  |  |
| **Follow-up time, mean, years (range)** | 7.8 (6.2–10.3) |  |  |
| **Fracture anatomy, n** |  |  |  |
| Fibular fracture | 39 | 14 | .7 |
| Medial malleolus fracture | 12 | 4 |  |
| Deltoid ligament rupture | 27 | 10 |  |
| Posterior malleolus fracture | 12 | 3 |  |
| Trimalleolar fracture | 8 | 3 |  |
| Medial malleolus fixation | 9 | 4 | .7 |
| Posterior malleolus fixation | 1 | 1 | .5 |
| **Syndesmosis fixation, n, (%)** | 39 | 14 |  |
| Syndesmosis screw fixation | 26 (67) | 6 (43) | .2 |
| Suture button fixation | 13 (33) | 8 (57) |  |
| *Patienst with syndesmosis fixation but lost to follow-up, excluded from the original trials and patients without WBCT  WBCT; weight-bearing cone beam computed tomography | | | |
