# Supplementary material for: Rotational Dynamics of the Distal Tibiofibular Joint After Operative Treatment of Ankle Fractures With Syndesmosis Injury
Source: Foot Ankle Int. 2025 Dec 8;47(2):216–23. doi: 10.1177/10711007251392222 (PMC12882989; doi:10.1177/10711007251392222)
Supplement: sj-docx-3-fai-10.1177_10711007251392222 – Supplemental material for Rotational Dynamics of the Distal Tibiofibular Joint After Operative Treatment of Ankle Fractures With Syndesmosis Injury [file sj-docx-3-fai-10.1177_10711007251392222.docx]

**Supplementary table 2.** Post hoc sample size and power calculations for main outcome variables.

|  | **Injured ankle**  **Mean (SD)** | **Non-injured ankle**  **Mean (SD)** | **Common SD for power calculation** | **N needed to show significance^1^** | **Current power^2^** |
| --- | --- | --- | --- | --- | --- |
| **Sagittal translation (mm)** | 1.3 (1.1) | 1.1 (1.0) | 1.1 | 475 | 0.122 |
| **Anterior width (mm)** | -0.7 (0.9) | -0.7 (0.8) | 0.9 | infinity | 0 |
| **Posterior width (mm)** | 1.0 (1.3) | 1.1 (1.0) | 1.2 | 2261 | 0.056 |
| **Tibiofibular clear space (mm)** | -0.1 (0.5) | 0.02 (0.7) | 0.6 | 535 | 0.114 |
| **Fibular rotation (degrees)** | 2.4 (4.1) | -0.2 (2.5) | 3.3 | 34 | 0.93 |
| ^1^N per group with alpha=0.05 and power=0.80  ^2^With alpha=0.05 and n=39 | | | | | |

Abbreviations: SD, standard deviation; N, number of patients
